# 43 Home alone: Factors associated with discharge with Home Health

**DOI:** 10.1093/jbcr/irad045.017

**Published:** 2023-05-15

**Authors:** Geraldine Nabeta, Barbara Okeke PhD, Carl Schulman, Louis Pizano, Joyce Kaufman, Shevonne Satahoo

**Affiliations:** St. George's University, Miami, Florida; St. George's University, Miami, Florida; University of Miami-Jackson Memorial Hospital, Miami, Florida; University of Miami, Miami, Florida; University of Miami, Miami, Florida; University of Miami, Miami, Florida; St. George's University, Miami, Florida; St. George's University, Miami, Florida; University of Miami-Jackson Memorial Hospital, Miami, Florida; University of Miami, Miami, Florida; University of Miami, Miami, Florida; University of Miami, Miami, Florida; St. George's University, Miami, Florida; St. George's University, Miami, Florida; University of Miami-Jackson Memorial Hospital, Miami, Florida; University of Miami, Miami, Florida; University of Miami, Miami, Florida; University of Miami, Miami, Florida; St. George's University, Miami, Florida; St. George's University, Miami, Florida; University of Miami-Jackson Memorial Hospital, Miami, Florida; University of Miami, Miami, Florida; University of Miami, Miami, Florida; University of Miami, Miami, Florida; St. George's University, Miami, Florida; St. George's University, Miami, Florida; University of Miami-Jackson Memorial Hospital, Miami, Florida; University of Miami, Miami, Florida; University of Miami, Miami, Florida; University of Miami, Miami, Florida; St. George's University, Miami, Florida; St. George's University, Miami, Florida; University of Miami-Jackson Memorial Hospital, Miami, Florida; University of Miami, Miami, Florida; University of Miami, Miami, Florida; University of Miami, Miami, Florida

## Abstract

**Introduction:**

Burn patients are discharged home with ongoing wound care needs. Many obtain assistance at home with home health services. There are few published studies that review factors associated with obtaining these services upon discharge. As such, this study aims to identify patient-specific and hospital-specific factors that may contribute to being discharged under the care of home health services.

**Methods:**

The National Inpatient Sample was queried for all patients with age ≥ 18 years with ICD-9 codes for total body surface area (TBSA) burn ≥ 20% and non-elective admissions. Patients were further selected for those discharge home (routine) and those discharged under care of organized home health service organization. Years included were 2013- third quarter of 2015. Age, race, percent TBSA burn, demographic data, hospital factors and mortality were recorded. Statistical analysis was done with Chi-Square testing. Discharge was then used to perform a binary logistic regression using the significant variables.

**Results:**

There were 3245 weighted cases. Females accounted for 21.3% of the population. Of these encounters, 22.8% were discharged home with home health services. The average age of those without home health was 39.61 ± 13.59 years, versus 44.75 ± 15.92 years for those with home health (p< 0.001). Gender, percent TBSA burn, quartile of median household income of residents in the patient's ZIP Code, patient insurance, use of alcohol or drugs (p< 0.001), hospital bed size, hospital region, and hospital control/ownership (p< 0.001) were found to be statistically significant on univariate analysis. Discharge with home health services was then used to perform a multivariate analysis with the statistically significant factors. This found age (p< 0.001), and alcohol or drug use (p< 0.001) to be significant factors. The additional statistically significant factors are listed in Table 1. Those with home health services were less likely to have history of drug or alcohol use (OR 0.550 [IQR 0.424-0.713]) but more likely to be older, and to have Government insurance (OR 2.899 [IQR 2.096-4.009]) or Private insurance (OR 2.398 [IQR 1.713-3.356]), compared to self pay. There is also significant variability based on hospital region.

**Conclusions:**

Multiple patient-specific and hospital-specific factors may influence the possibility of burn patient being discharged home with home health services. It appears that patients who may benefit from these services were in fact captured as higher rates of home health services were noted among those with government insurance and within the lowest quartile of income based on patient zip codes. However, there seems to be room for improved services across the nation as there is great variability based on region.

**Applicability of Research to Practice:**

Discuss factors associated with patients being discharged home with home health services.

Identify areas of possible improvement to maximize access to these services for all burn patients.

